# How is your mindfulness?: Data on qualitative interpretations of the meaning of mindfulness

**DOI:** 10.1016/j.dib.2019.104232

**Published:** 2019-07-06

**Authors:** Ying Hwa Kee, Chunxiao Li

**Affiliations:** Nanyang Technological University, Singapore

**Keywords:** Mindfulness, Mental skills, Adults, Interpretation, Wellbeing

## Abstract

Despite the growing interest in mindfulness over the last two decades, what mindfulness means to people can be different due to diversity in personal experience, social, and cultural background. To gain some insights on what mindfulness means to different people in different countries/regions, a research project entitled “How is your mindfulness” was conducted in early 2019. 247 adults from 35 countries/regions provided valid responses to an online questionnaire. Primarily, the questionnaire solicited open-ended responses about participants’ interpretation of mindfulness and descriptions of their own their mindfulness skills. Participants also rated their mindfulness skill, self-esteem, and life satisfaction through single-item scales. Availability of the open-ended responses is valuable for researchers interested in further understanding mindfulness from a qualitative angle. The availability of data from single-item measures of mindfulness skills, self-esteem and life esteem also provides a quantitative dimension to this issue. Taken together, the current data set provides an initial record of how different people are interpreting mindfulness, and could be further developed to advance mindfulness research.

**Table:** Specifications table

Subject area	*Psychology*
More specific subject area	*Mindfulness*
Type of data	*Data spreadsheet*
How data was acquired	*Online survey questionnaire*
Data format	*Processed*
Experimental factors	*Online responses were solicited through various social media platform (e.g. Twitter, Facebook, online forum) and through emails between 14 Jan 2019 to 28 Feb 2019. Only participants of 21 years or above are eligible. Participants regardless of mindfulness experience are eligible.*
Experimental features	*464 responses were logged. After removing invalid cases (e.g. incomplete responses, spam), 247 responses were retained.*
Data source location	*Singapore (n = 114); United States (n = 49); United Kingdom (n = 16); Australia (n = 13); Canada (n = 9); France (n = 6); Netherlands (n = 4); Norway (n = 3); Brazil (n = 2); Denmark (n = 2); Germany (n = 2); Hungary n = (2); Malaysia (n = 2); Taiwan(n = 2); Austria (n = 1); Belgium (n = 1); Croatia (n = 1); Estonia (n = 1); Finland (n = 1); Greece (n = 1); Hong Kong (n = 1); India (n = 1); Indonesia (n = 1); Ireland (n = 1); Japan (n = 1); Kuwait (n = 1); Lebanon (n = 1); Macedonia (n = 1); Panama (n = 1); Poland (1); Romania (n = 1);* *Russian Federation (n = 1); Spain (n = 1); Sweden (1); and Switzerland (n = 1)*
Data accessibility	https://doi.org/10.25340/R4/BIOIZO
Related research article	*Kee, Y.H. Reflections on athletes' mindfulness skills development: Fitts and Posner's (1967) three stages of learning. J. Sport Psychol.* *Action. (In press).*https://doi.org/10.1080/21520704.2018.1549640

**Table:** 

**Value of the data** • Interest and research in mindfulness has grown exponentially over the past two decades given its salutary effects [1]. However, the interpretation of what mindfulness means remains relatively diverse among scholars and the general population. Thus, there is a need to continual capture the nuances of what people interpret mindfulness to be to support the development of mindfulness research and mindfulness skill [2]. • The data is the outcome of an online survey which solicits participants' description of what mindfulness mean to them and their own mindfulness skill, as well as responses to self-rating of mindfulness skill, self-esteem and life-satisfaction. • Participants' open-ended responses on mindfulness provide valuable insights about current diverse perspectives of mindfulness. Further analysis undertaken by researchers could lead to fresh interpretations of what mindfulness mean within the scholastic space, supporting further development of new research directions in mindfulness research. • Quantitative data based on single-item self-rating of mindfulness skill, life-satisfaction [3] and self-esteem [4] provides for researchers additional markers to examine relationships between these three variables and the qualitative data.

## 1 Data

A wide-formatted data set in comma separated format (csv) format comprised of data points from 247 participants across 35 locations internationally. The variable headings (description, if any, in parentheses) are as follows: SN (Serial number), Start date, End date, Duration (Duration taken to complete survey in seconds), Age, Gender, Describe.Mindfulness (To you, what does mindfulness mean?), Own.Mindfulness (How is your mindfulness skill?), Rate.Mindfulness (single item self-rating of mindfulness skill), Life satisfaction (single item rating of life satisfaction [3]), Self-esteem (single item rating of self-esteem [4]), Location (derived based on IP addresses logged), and About (additional comments given by the participants).

## 2 Experimental design, materials, and methods

The Institutional Review Board at the Nanyang Technological University, Singapore approved this research (IRB-2018-12-015).

An online survey comprised of the items depicted in Appendix A was made available through Qualtrics between 14 January 2019 to 28 February 2019. The survey items include age, gender, descriptions of what mindfulness means for them, description of their mindfulness skills, self-rating of mindfulness skills, life satisfaction [3], and self-esteem [4]. Location information was derived from the IP addresses logged.

To solicit responses from an international sample, the link to the survey was disseminated through various social medial channels such as Twitter, Facebook, Reddit, online forum, and through emails. The targeted audience were participants of 21 years or above regardless of mindfulness experience. Participants provided informed consent online. Only participants who expressed willingness to have their data published in the public domain are included. No names or personal identifier was collected. Eventually, a total of 464 responses were logged. After removing invalid cases (e.g. incomplete responses, spam), 247 responses were retained and presented in this data paper.

## Appendix A Supplementary data

The dateset can be found at https://doi.org/10.25340/R4/BIOIZO. The following is the questionnaire set linked to this article:
