# Supplementary material for: How is your mindfulness?: Data on qualitative interpretations of the meaning of mindfulness
Source: Data Brief. 2019 Jul 6;25:104232. doi: 10.1016/j.dib.2019.104232 (PMC6656984; doi:10.1016/j.dib.2019.104232)
Supplement: Supplementary file 1 [file mmc1.pdf]

Appendix A

Welcome to the research study "How is your mindfulness skill?".

We are interested in understanding "Mindfulness Skills" as perceived by people across the world.

Anyone above 21 of age, regardless of prior experience in mindfulness practice, is welcomed to contribute to this survey. We hope to receive responses from individuals with different countries, background and mindfulness experience. The responses will be primarily analysed using text-mining approaches. Findings will be shared with the scientific community in due course.

This survey comprises 8 questions, of which 3 are open-ended questions. Primarily, you will be presented with a common explanation of mindfulness, and some questions on your views and experience about mindfulness skills. Questions on your basic demographics info, self-esteem and life satisfaction are also asked.

Your participation in this study is anonymous, except that information regarding your IP address and approximate location will be logged by the system. Time taken to respond to some of the questions is also recorded.

This is a short study and should take you around 1 to 5 minutes to complete, and you will receive no incentive for your participation. Your participation in this research is voluntary. You have the right to withdraw at any point during the study, for any reason, and without any prejudice.

By clicking the first button below, you acknowledge that your participation in the study is voluntary, you are at least 21 years of age, and that you are aware that you may choose to terminate your participation in the study at any time and for any reason.

At the end of the study, you will also be asked if you agree to include your data (without your IP address) in our open and public sharing of data gathered through this project.

The survey will end on 28 February 2019. Please only attempt the survey only once to help us maintain the validity of findings.

If you would like to contact the Principal Investigator in the study to discuss this research, please e-mail Dr. KEE Ying Hwa at yinghwa.kee@nie.edu.sg.

□ I consent, begin the study

□ I do not consent, I do not wish to participate

1. Age?

□ 21 - 24

□ 25 - 34

□ 35 - 44

□ 45 - 54

□ 55 - 64

□ 65 - 74

□ 75 - 84

□ 85 or older

2. Gender?

□ Male

□ Female

□ Others

3. One way of describing "Mindfulness" is to refer to it as a mental skill of **purposefully paying nonjudgmental attention to what is happening at the present moment in a repetitive fashion**.

To you, what does "Mindfulness" skill mean?

4. To what extent do you find it EASY to use mindfulness skills in everyday life?

□ 9 - Very Easy

□ 8

□ 7

□ 6

□ 5

□ 4

□ 3

□ 2

□ 1 - Very Hard

5. How would you describe your own mindfulness skill?

6. All things considered, how satisfied are you with your life as a whole?

□ 10 - Completely satisfied

□ 9

□ 8

□ 7

□ 6

□ 5

□ 4

□ 3

□ 2

□ 1 - Not at all satisfied

7. Please indicate to what extent the following statement applies to you: “I have high self-esteem.”

□ 5 - Very true of me

□ 4

□ 3

□ 2

□ 1 - Not very true of me

8. If you like to describe yourself further, feel free to do so in the space below. Be careful not to leave personal information like your name and contact information.

9. You have completed all the survey questions. Before we end, we like to know if you allow us to include your data (excluding your IP address) in our public sharing of raw data collected from this study.

□ Agree

□ Disagree
